# High incidence of pulmonary tuberculosis in children admitted with severe pneumonia in Uganda

**DOI:** 10.1186/1471-2431-13-16

**Published:** 2013-01-31

**Authors:** Josephine M Nantongo, Eric Wobudeya, Ezekiel Mupere, Moses Joloba, Willy Ssengooba, Harriet N Kisembo, Irene R Lubega, Philippa M Musoke

**Affiliations:** 1 Department of Paediatrics & Child health, Makerere University College of Health Sciences, Kampala, Uganda; 2 Directorate of Paediatrics & Child Health, Mulago National Referral Hospital, Kampala, Uganda; 3 Department of Microbiology, Makerere University College of Health Sciences, Kampala, Uganda; 4 Makerere University-Johns Hopkins University Research Collaboration, Kampala, Uganda; 5 Radiology, School of Medicine, Makerere University College of Health Sciences, Kampala, Uganda

## Abstract

**Background:**

A high prevalence of tuberculosis (TB) in children presenting with severe pneumonia has previously been reported in South Africa. However, little is known about TB among children with pneumonia in Uganda and other resource limited countries. Moreover, TB is associated with high morbidity and mortality among such children. We conducted this study to establish the burden of pulmonary TB in children admitted with severe pneumonia in our setting.

**Methods:**

A cross-sectional study was conducted at Mulago, a National Referral and teaching hospital in Uganda. Hospitalised children 2 months to 12 years of age with severe pneumonia based on WHO case definition were enrolledfrom February to June 2011. Children with a previous TB diagnosis or receiving anti-TB treatment were excluded. Each child was screened for TB using Tuberculin skin test, Chest X-ray, induced sputum samples and blood culture for mycobacterium. Sputum smears were examined using fluorescent microscopy, and cultured on both Lowenstein Jensen media (LJ) and Mycobacterial Growth Indicator Tubes (MGIT).

**Results:**

Of the 270 children with severe pneumonia who were recruited over a 5-month period in 2011, the incidence ratio of pulmonary TB in children admitted with severe pneumonia was 18.9% (95% CI 14.6 – 23.9). The proportion of culture confirmed PTB was 6.3% (95% CI 3.8 – 9.7). Age group under 1 year and 1 to 5 years (OR 2.8 (95% CI 1.7 – 7.4) and OR 2.4 (95% CI 1.05 – 5.9) respectively) were more likely to be associated with pulmonary TB compared to those children over 5 years of age. A history of TB smear positive contact was associated with pulmonary TB (OR 3.0 (95% CI 1.3–6.5).

**Conclusions:**

We found a high burden of pulmonary TB in children admitted with severe pneumonia. These data highlight the need for TB screening in children admitted with severe pneumonia so as to improve TB case finding and child survival.

## Background

Tuberculosis (TB) is a global problem with about 9 million incident cases in 2010 of whom children under 15 years accounted for about 10% [1]. Annually, an estimated 400,000 deaths in children are associated with TB. Some of these deaths may be due to delay in diagnosis since paediatric TB sometimes presents as an acute pneumonia [2]. Globally, the pneumonia mortality accounts for about 19% of deaths in children and 70% of these deaths occur in sub-saharan Africa and East Asia [3]. In Uganda, pneumonia contributes 21% of the under five mortality ( U5M ) [4]. TB in children sometimes presents as acute pneumonia [5] and TB is one of the leading causes of morbidity and mortality across all age groups throughout the world, especially in developing countries [6,7]. In infants TB commonly manifest with signs and symptoms of acute pneumonia [8] that may lead to delay in diagnosis, prolonged hospital stay and increased mortality. The diagnosis of TB in children still remains a challenge and the proportion diagnosed due to non-response to antibiotics represents children with delayed diagnosis of TB [9]. The need to increase TB detection and reduce on the missed opportunities for early diagnosis of TB in high risk populations that include children has been highlighted by the WHO/TB STOP partnership strategy for TB through intensified case finding [10]. An understanding therefore, of the burden of TB in children admitted with severe pneumonia will not only heighten the index of suspicion but will also call for the extension of the policy on intensified case finding to pneumonia cases. An exploration of the clinical characteristics associated with TB in children with severe pneumonia would be useful. This study aimed to establish the incidence ratio of pulmonary TB and describe the factors associated with pulmonary TB in children hospitalised with severe pneumonia at a paediatric emergency unit.

## Methods

### Study design

This was a cross sectional analytical study.

### Setting

The study was done at the Mulago National Referral Hospital in Kampala, the capital city with a day population of 2.5 million, HIV sero-prevalence of 8.5% among adults, HIV sero-prevalence of 2.2% in children under five and BCG vaccination coverage of 94.6%. This hospital with a 1700-bed capacity and an annual turnover of 100,000 patients also serves as the primary health care facility for the surrounding areas. The children were recruited from the paediatric emergency unit between February and June 2011. This paediatric emergency unit has an annual turnover of 20,000 patients with pneumonia admissions accounting for about 20% of the admissions.

### Participants and procedures

Children aged 2 months -12 years who fulfilled the WHO case definition for severe or very severe pneumonia and whose caretaker gave informed consent were eligible to participate in the study. The children above 8 years of age provided assent. Children already on treatment for TB were excluded. The enrolment was consecutive after at least a 24-hour admission period to limit the misclassification of the cases that fulfil the WHO criteria for severe pneumonia but are not pneumonia for example asthma and severe anaemia.

Severe pneumonia was defined as a presentation with cough or difficulty in breathing, fast breathing and chest in drawing. A very severe pneumonia case fulfilled the case definition for severe pneumonia plus either cyanosis, convulsions or inability to feed [11]. The potential participants were identified at the triage by the triage nurse or the research assistant.

After obtaining informed consent, specific clinical history that included duration of cough, TB contact, weight loss, BCG vaccination, previous pneumonia episodes was obtained from the caregiver or parent. The children were examined for BCG scar, peripheral lymphadenopathy and their weights and height obtained. The specific investigations done included; Full blood count, blood culture for mycobacterium, one sputum induction, tuberculin skin test (TST) placement by the Mantoux method and chest radiography. The HIV serology results were obtained from the Hospital Routine HIV counselling and testing (HCT) register. The caregivers or parents were encouraged to have the children tested for HIV within the hospital HCT program. The children found to be HIV positive were linked to the HIV care centre at the Mulago Hospital for further management. The Chest radiography and placement of TST were done on the day of enrolment. The TST was read within 48 – 72 hours by the same trained nurse who placed the skin test. The one sputum induction was done early in the morning within 24 hours of admission. The older children provided at least 2 expectorated sputum samples. The sputum samples were placed in a cool box with ice packs and taken to the - Mycobacteriology Laboratory, Department of Microbiology, Makerere University College of Health sciences for processing within 1 hour of collection.

### Laboratory investigations

Complete blood count was done using a coulter Act Diff 5 machine. Blood (2-5 ml) for Mycobacterial culture was aseptically inoculated into BD BACTEC-MYCO/F-Lytic media (Becton and Dickson, Franklin Lakes, NJ USA) for up to six weeks at the Mycobacterialogy (BSL-3) laboratory. Cultures in which the machine flagged positive were unloaded and a Ziehl Nelseen (ZN) smear for Acid Fast Bacilli (AFB) was done. A portion was also inoculated onto a Blood Agar Plate (BAP) to rule out contaminants. Cultures that revealed AFB on ZN smear were subjected to Capilia TB NeoTM (TAUN, Numazu, Japan) for identification of *Mycobacterium tuberculosis* complex (MTBC). However, cultures which were ZN negative and revealed growth on BAP were considered contaminated.

Sputum samples were prepared for culture according to laboratory standard operating procedures [12]. A volume 0.5 ml of the re-suspended deposit was inoculated in a BACTEC BBL MGITTM 960 tube (Becton and Dickson, Franklin Lakes, NJ USA). Additionally, two drops were inoculated in LJ tubes (Becton and Dickson, Franklin Lakes, NJ USA) and cultured. A smear for fluorescent microscopy was also done according to standard procedures [13].

Cultures were incubated at 37°C for 6 weeks in MGIT and 8 weeks on LJ media. For purity, any MGIT-positive culture was sub-cultured at 37°C on blood agar for 24 hours and a Ziehl Neelsen (ZN) smear done. Cultures which were ZN-positive and pure (i.e. no growth on blood agar) were subjected to Capilia TB NeoTM (TAUN, Numazu, Japan) for identification of the MTB complex [14]. Cultures with growth on blood agar but also ZN-positive were sub-cultured again as described above; the persistent ZN-negative cultures with growth on blood agar were considered contaminated. All ZN-positive, blood agar negative and Capilia TB NeoTM negative samples were considered as having Mycobacteria Other Than Tuberculosis (MOTT). Otherwise absence of colonies on LJ or fluorescence on MGIT was considered negative. The data were entered into a computerized laboratory access database linked with patient records on sample reporting form.

The main outcome measure was the diagnosis of pulmonary TB. A case of Pulmonary TB is one that fulfilled either the confirmed case or probable case definition [15]. A confirmed case is one with culture confirmed MTBC while a probably Pulmonary TB case is one with at least one of the signs and symptoms suggestive of TB AND Chest radiography consistent with intrathoracic disease due to MTBC AND there is at least one of the following; Documented exposure to MTBC infection, Immunological evidence of MTBC infection. The chest radiography reading for features of TB was done by 2 independent blinded radiologists with the third acting as a tie-breaker.

The study was approved by the School of Medicine Research and Ethics committee, Makerere University College of Health Sciences. Written informed consent was given by the parents/caretakers if they agreed to their children participating in the study and assent was obtained from older children. Children that were diagnosed with Pulmonary TB were linked to the National TB and Leprosy control program for care and treatment.

### Statistical issues

Using the Kish-Leslie formula, a minimum sample size of 196 participants was sufficient to determine the burden of Pulmonary TB among children admitted with severe pneumonia while a minimum sample size of 257 with at least 43 TB cases was sufficient to explore associated factors.

The data was captured using Epidata version 3.1 and analyzed using SPSS (version 14.0). The proportion with Pulmonary TB was determined with its 95% CI. The continuous variables were summarized as means (SD) or medians (IQR). Any associations with Pulmonary TB for categorical variables were explored using the χ^2^ test whereas normally distributed continuous variables were tested using student’s t-test and skewed data using the Mann-Whitney U test. Multivariate analysis using logistic regression was carried out on variables with p- value of less than 0.2 at bivariate to adjust for confounding. P value <0.05 was considered significant.

## Results

### Participant descriptive statistics

**Figure 1:**
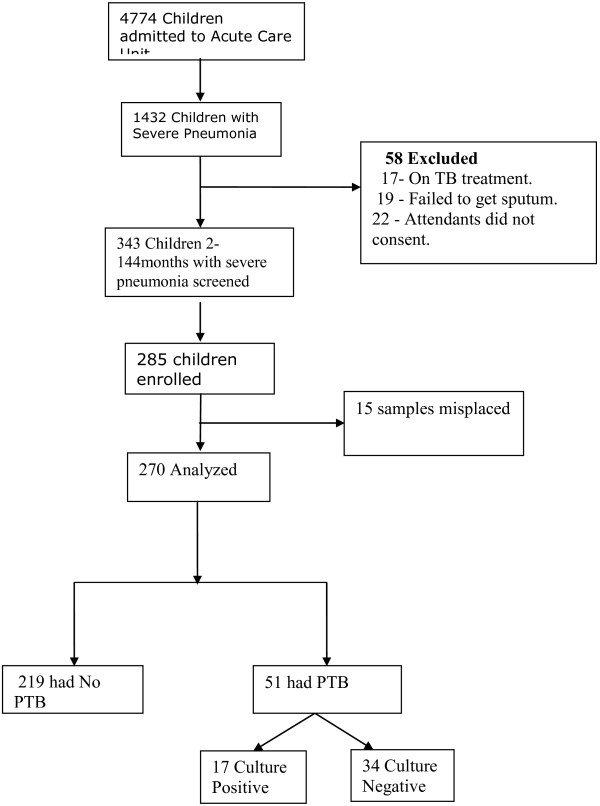
Study participant recruitment flow chart.

A total of 285 children with severe or very severe pneumonia were enrolled and 270 were included in the analysis (Figure 1). The median age was 15 months (IQR 7–36) with 85.5% of the study participants under 5 years of age. The HIV seroprevalence was 15.2% and 0.7% were HIV exposed but uninfected. Forty one children were HIV positive. Fourteen had PTB and 27 had No TB. Only 1 of the 14 TB cases was on ARVs while 6 without TB were on ARVs. None was on isonaizide prophylaxis. The majority, 93% (251/270), of caretakers reported BCG immunisation with 75.3% (203/270) of the children having a BCG scar. The WBC were similar; 13,680 in PTB Vs 14, 299 in No PTB, p = 0.8, as well as the Lymphocytes; 6567 in PTB Vs 7178 in No PTB, p=0.4. Other baseline characteristics are shown in Table 1.

**Table 1:** Baseline characteristics of children admitted with severe pneumonia (N=270)

	***Age group***		
	**Under 1 year**	**1 – 5 years**	**Above 5 years**
	**(n=101)**	**(n=130)**	**(n=39)**
**Cough > 2 weeks**	34 (29.8%)	57 (50%)	23 (20.2%)
**History of weight loss**	49 (33.8%)	66 (45.5%)	30 (20.2%)
**Peripheral lymphadenopathy**	11 (22%)	28 (56%)	11 (22%)
**History of TB Contact**	11 (22.9%)	31 (64.6)	6 (12.5%)
**Previous pneumonia**	26 (24.1%)	56 (51.9%)	26 (24.1%)
**Moderate/severe malnutrition**	22 (31.4%)	37 (52.9%)	11 (15.7%)
**HIV positive**	12 (29.3%)	21 (51.2%)	8 (19.5%)

### Incidence of pulmonary TB in children admitted with severe pneumonia

Of the 270 children, 18.9% (95% CI 14.6 – 23.9) were diagnosed with Pulmonary TB. The culture confirmed cases were (17/270) 6.3% (95% CI 3.8 – 9.7) while the probable cases were 12.6% (95% CI 9.0–17). Two children (5 months and 8 years) had mycobacteremia while one case (11 years) had both Pulmonary TB and mycobacteremia. The remaining 14 had MTB confirmed from sputum cultures. Forty seven percent (24/51) of the children with PTB were under 2 years of age. Thirty four percent of the HIV positive children had pulmonary TB and 27.5% of the children with TB were co-infected with HIV. The BCG scar was absent in 35.3% of the children with TB compared to 26.9% of those without TB, the difference was not statistically significant (p value = 0.2). Abnormal CXRs were found in (45/51) 88% of PTB cases Vs (136/219) 62% of None PTB cases OR 4.6 (1.8 – 11.2), p < 0.001.

### Factors associated with PTB in children admitted with severe pneumonia

In children with severe pneumonia, the strongest clinical associations with Pulmonary TB were; history of cough > 2 weeks, history of weight loss, history of contact with PTB, age under 2 years, significant peripheral lymphadenopathy and HIV positive status (Table 2). On stratifying by HIV status (data not shown), there was no association between pulmonary TB and severe pneumonia in the HIV positive children except for the history of contact. In the HIV negative children, age under 2 years, history of contact and peripheral lymphadenopathy remained associated with Pulmonary TB at univariate analysis. Further comparison of some characteristics between the culture confirmed and culture negative PTB cases revealed no difference (Table 3).

**Table 2:** Comparison of participant characteristics with and without PTB admitted with severe pneumonia (N=270)

***Variable***	***PTB***	***No PTB***	***OR (95% CI)***	***p –value***
	***n=51 (%)***	***n=219 (%)***		
**History of cough**				
>2 weeks (42.2%)	32 (62.7)	82 (37.4)	2.8 (1.4 - 5.2)	0.001
<2 weeks (57.8%)	19 (37.3)	137 (62.6)		
**History of weight loss**				
Present (18.5%)	17 (33.3)	33 (15.1)	2.8 (1.4 - 5.6)	0.002
Absent (81.5%)	34 (66.7)	186 (84.90)		
**Peripheral lymphadenopathy**				
Present (18.5%)	16 (31.4)	34 (15.5)	2.4 (1.2 - 4.9)	0.009
Absent (81.5%)	35 (68.6 )	185 (84.5)		
**Malnutrition (WHZ)**				
Moderate/severe (26%)	18 (35.3)	52 (23.7)	1.7 (0.9 - 3.3)	0.23
None/mild (74%)	33(64.7)	167 (76.3)		
Anaemia*				
Mod/severe (69.6%)	42 (82.4)	146 (66.7)	2.3 (1.07 - 5.0)	0.028
None/mild (30.4%)	9 (17.6)	73 (33.3)		
**HIV serology**				
Positive (15.2%)	14 (27.5)	27 (12.3)	2.6 (1.2 - 5.6)	0.007
Negative (84.8%)	37 (72.5)	192 (87.7)		
**TB contact**				
Yes (17.8%)	19 (37.3)	29 (13.2)	3.89 (1.9 - 7.7)	<0.001
No (82.2%)	32 (62.7)	190 (86.8)		
**BCG immunisation**				
Yes (93%)	43 (84.3)	208 (95)	0.28 (0.1 - 0.74)	0.007
No (7%)	8 (15.7)	11 (5.0)		
**Age group**				
Under 1 year	13 (25.5)	88 (40.2)		0.008
1 – 5 years	24 (47.1)	106 (48.4)		
Above 5 years	14 (27.5)	25 (11.4)		

Definitions: WHZ, < -2 Z score – severe/moderate malnutrition. Z scores obtained using EpiInfo 3.5.1 Nutrition program. Anaemia, mod/severe, < 7 gm/dl for age group less than 5 years OR < 8 gm/dl for age group 5 – 14 years.

**Table 3:** Comparison of some characteristics of confirmed and culture negative PTB cases admitted with severe pneumonia (N=51)

***Variable***	***Confirmed PTB***	***Culture negative PTB***	***OR (95% CI)***	***p –value***
	***n=17 (%)***	***n=34 (%)***		
**History of cough**				
>2 weeks (63%)	12 (71)	20 (59)	1.6 (0.4 - 5.8)	0.41
<2 weeks (37%)	5 (29)	14 (41)		
**History of weight loss**				
Present (59%)	11 (65)	19 (56)	1.4 (0.4 - 4.8)	0.5
Absent (41%)	6 (35)	15 (44)		
**History of wheezing**				
Yes (12%)	3 (18)	3 (9)	2.2 (0.3 - 12.3)	0.3
No (88%)	14 (82)	31 (91)		
**Peripheral lymphadenopathy**				
Present (31%)	5 (29)	11 (32)	0.8 (0.2 - 3.0)	0.8
Absent (69%)	12 (71 )	23 (68)		
**Malnutrition (WHZ)**				
Moderate/severe (35%)	7 (41)	11 (32)	1.4 (0.4 - 4.8)	0.5
None/mild (65%)	10(59)	23 (68)		
Anaemia*				
Mod/severe (82%)	14 (82)	28 (82)	1 (0.2 - 4.6)	>0.999
None/mild (18%)	3 (18)	6 (18)		
**HIV serology**				
Positive (28%)	3 (18)	11 (32)	0.4 (0.1 - 1.8)	0.2
Negative (72%)	14 (82)	23 (68)		
**TB contact**				
Yes (37%)	6 (35)	13 (38)	0.8 (0.2 - 2.9)	0.8
No (63%)	11 (65)	21 (62)		
**Reduced activity**				
Yes (67%)	13(76.5)	21(62)	2.0 (0.5 – 7.5)	0.2
No (33%)	4(23.5)	13(38)		
**CXR**				
Abnormal (41%)	7 (41)	14 (41)	1 (0.3 – 3.2)	>0.999
Normal (59%)	10 (59)	20 (14)		
**BCG immunisation**				
Yes (84%)	14 (82)	29 (85)	0.8 (0.1 - 3.8)	0.7
No (16%)	3 (18)	5 (15)		
**Age group**				
Under 1 year (25.5%)	6 (35)	7 (21)		0.4
1 – 5 years (47%)	6 (35)	18 (53)		
Above 5 years (27.5%)	5 (29)	9 (26)		

At multivariate analysis, the factors that remained independently associated with pulmonary TB were age under five years and history of smear positive TB contact (Table 4).

**Table 4:** Multivariate analysis of factors independently associated with pulmonary tuberculosis in children admitted with severe pneumonia

***Variable***		***OR (95% CI)***	***p-value***
**Age group (months )**	Above 5 years	1	
	Under 1 year	2.8 (1.1 - 7.4)	**0.028**
	1- 5 years	2.4 (1.05 - 5.9)	0.038
**Cough for 2 or more weeks**		1.7 (0.8 - 3.4)	0.11
**History of TB contact**		3.0 (1.3 - 6.5 )	**0.005**
**Peripheral lymphadenopathy**		1.7 (0.7 - 3.7)	0.17
**Positive HIV serology**		1.3 (0.8 - 2.0 )	0.15
**Mod/severe Anaemia**		1.9 (0.8 - 4.4)	0.12

## Discussion

We have shown that the incidence of PTB in children admitted with severe pneumonia is high with about one in five case of severe pneumonia also having pulmonary TB. This result is important since pneumonia is a major contributor to hospital admissions in most resource limited settings and concomitant TB has been associated with poor outcomes in both adults and children [5,16]. To the best of our knowledge, this is the first study done in Uganda to find the burden of pulmonary TB in children admitted with severe pneumonia. High rates of pulmonary TB have been reported elsewhere in children failing on pneumonia treatment [5]. The TB rates are greatly influenced by the sensitivity and specificity of the signs and symptoms employed to select the participants. A study conducted in our setting using the probable case definition as the entry point for further evaluation of pulmonary TB found a prevalence of 30% among children attending the emergency paediatric department [17] compared to our finding of 19% in children admitted with acute pneumonia. There are several studies conducted in South Africa that have investigated PTB in children with acute lower respiratory tract infections [5,18,19]. Moore D et al reported a similar finding in a pneumococcal conjugate vaccine probe study where 18.5% of the episodes of lower respiratory infections investigated had pulmonary TB [18]. Our study compares well with this vaccine probe study [18] that was conducted in the era of anti-retroviral therapy (ART) availability and similar study setting except that we investigated only severe pneumonia cases. The proportion of confirmed MTB cases of 6.3% in our study was similar to that reported by Moore D et al [18] of 6.7% but differs from that of 15% by McNally et al [5]. This difference between McNally and our study may be explained by the fact that they studied a population of treatment failures where the likelihood of PTB is much higher. An alternative explanation could be the much higher background HIV sero-prevalence of 36.5% at the time of their study when there was no ready access to ART compared to our background sero-prevalence rate of 8.5% with ready access to ART. Among the HIV positive children, the proportion of culture confirmed TB was 7.3% which is similar to a prevalence of 8% found by Madhi SA. et al from South Africa [19]. Our study set out to determine the incidence of PTB defined as probable or culture confirmed PTB rather than culture confirmed PTB alone in children admitted with severe pneumonia.

In our study, children with a history of a smear positive TB contact in the past one year were three times more likely to have pulmonary TB. A community study in south Africa found a five-fold increased TB burden in children with recent household TB contact [20]. Similar findings have consistently been reported in several other studies [17,21,22]. Age below 5 years and history of TB contact were independently associated with pulmonary TB in children admitted with severe pneumonia. In our study, Children under 1 year and those between 1 and 5 years were about two to three times more likely to have pulmonary TB compared to those above five years. This finding is a reflection of the epidemiological distribution of TB between 0 and 14 years where more TB is observed in under fives compared to the above five years age group [20].

Our findings therefore underscore the need for extending the intensified case finding approach to include children admitted with severe lower respiratory infections. This strategy is likely to increase paediatric TB case detection and reduce on the missed opportunities for early diagnosis of TB in children. Control of TB in children has been given little attention because most children are sputum smear negative and previously only sputum smear positivity has been used for the estimates of the magnitude of TB [23]. Our study’s finding of a large number of culture proven TB in children with severe pneumonia aged under five years calls for greater emphasis on routinely performing sputum inductions and cultures in children.

Reducing the burden of TB in children in our setting will require changing sputum examination policies to have all sputum samples from children undergo culture. There is need to improve early and proper diagnosis, early treatment, contact tracing, and correct reporting of paediatric TB. This commitment will require allocation of additional resources for TB control [24] with increased attention to paediatric TB.

This study was conducted in the emergency paediatric unit where the children with severe pneumonia would be captured. The study setting is similar to most health facilities in Uganda. We believe this study can be generalised to the emergency paediatric units within Uganda.

This study is limited by the inherently low yield rates using the available diagnostic techniques due to the paucibacillary nature of TB in children. The TB culture that was the gold standard for confirmed TB in this study has a yield rate of 30-40% [25]. We used the clinical criteria that included both the confirmed and probable cases in attempting to address this limitation. We excluded 34 cases either at analysis or screening due to either failure to obtain samples or lost results. If half of these results were positive for MTB, this may have a modest influence on our results either upwards or downwards. However, given the known low MTB culture yield from childhood samples, we believe our results are an acceptable reflection of the burden of PTB in severe pneumonia cases in our setting.

## Conclusions

The incidence of pulmonary TB in children admitted with severe pneumonia in Mulago Hospital is high. The children with severe pneumonia aged below five years with a recent smear positive TB contact are more likely to have pulmonary TB when admitted with severe pneumonia. Further prospective research is required to get a better understanding of the predictors of pulmonary TB focusing on children of five years and below with severe pneumonia.

## Competing interest

The authors declare that they have no competing interests.

## Authors’ contributions

JN: conceived the idea and implemented the study. She was the principle investigator for this work. She prepared the primary manuscript. EW and EM contributed in the refining of the idea, designing of the study, planning the statistical analysis, writing and reviewing of the manuscript. MJ, WS: Besides ensuring laboratory quality control for TB specimens, contributed to writing and reviewing of the manuscript. HK: participated in reading the chest radiographs, contributed to writing and reviewing the manuscript. LIR, PM: contributed to the writing and review of manuscript. All the authors read and approved the final manuscript.

## Pre-publication history

The pre-publication history for this paper can be accessed here:

http://www.biomedcentral.com/1471-2431/13/16/prepub
